# CANTOS: A breakthrough that proves the inflammatory hypothesis of atherosclerosis

**DOI:** 10.21542/gcsp.2018.2

**Published:** 2018-03-14

**Authors:** Mohamed Hassan

**Affiliations:** Cardiology Department, Cairo University, Cairo, Egypt; Cardiology Department, Aswan Heart Centre, Aswan, Egypt

## Abstract

Atherosclerosis is no longer considered solely a disorder of subintimal deposition of modified low-density lipoprotein particles in the arterial wall. Rather, it is known to be a chronic inflammatory disorder. No evidence has shown that reducing vascular inflammation in the absence of concomitant lowering of lipoproteins levels reduces the rates of adverse cardiovascular (CV) events. Canakinumab, a fully human monoclonal antibody that neutralizes interleukin (IL)-1β, significantly reduced the rate of recurrent CV events in patients with prior myocardial infarction in the Canakinumab Anti-inflammatory Thrombosis Outcome Study (CANTOS). Canakinumab has no effect on CV or all-cause mortality, however it was associated with high incidence of fatal infections. Thus, the net benefit needs to be properly addressed in future studies that evaluate the potential benefit of the anti-inflammatory therapies and whether it can change clinical practice in the near future.

## Introduction

**Figure 1.**
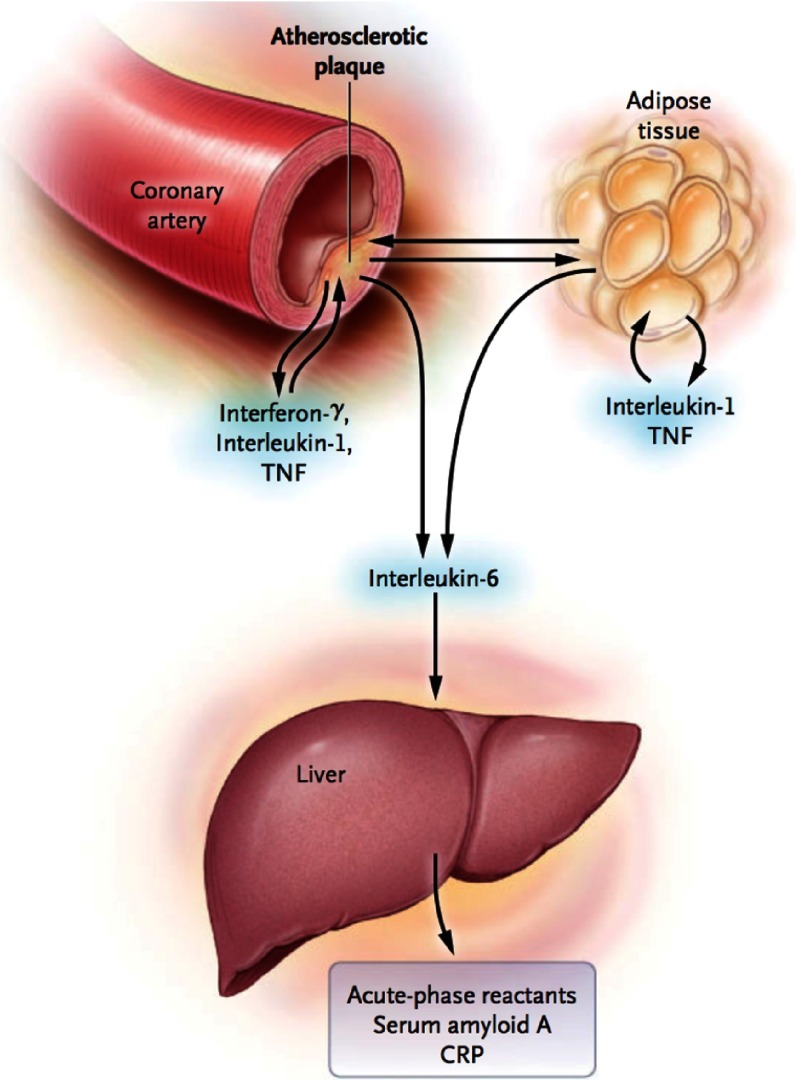
Cytokines cascade. Adapted from reference [2].

Inflammation plays a crucial role in all stages of the atherothrombotic process, in which immune mechanisms interact with metabolic risk factors to initiate, propagate, and activate atherosclerotic lesions in the arterial tree^1–3^. Activated immune cells – macrophages and T1 helper cells – in the plaque produce many inflammatory cytokines, such as interferon-γ, interleukin (IL)-1, and tumor necrosis factor (TNF-α), which subsequently induce the production of substantial amounts of IL-6 (Figure 1)^3^. IL-6, in turn, stimulates the production of large amounts of acute-phase reactants, including C-reactive protein (CRP), serum amyloid A, and fibrinogen, especially in the liver.

**Figure 2.**
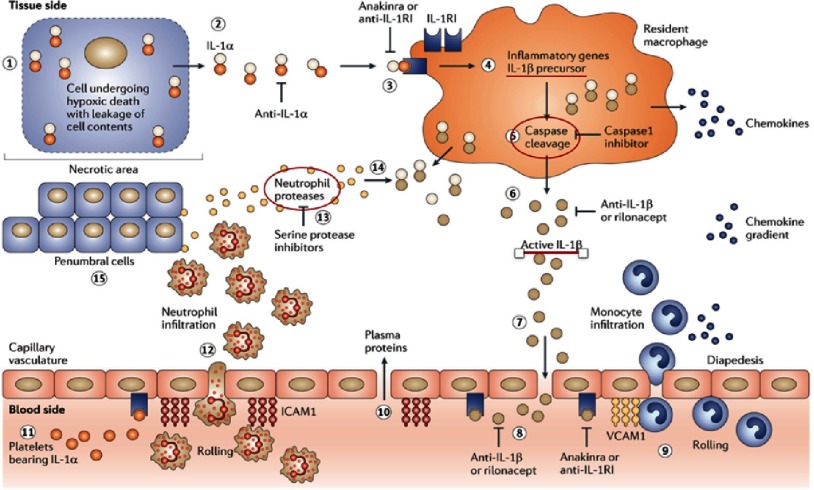
Role of IL-1β in inflammation. Macrophages synthesize IL-1β precursor in response to activation by IL-1α. The IL-1β precursor undergoes either intracellular processing by caspase 1 or extracellular processing by serine proteases -released from neutrophils- to produce active IL-1β. Active IL-1β breaks the vascular integrity to gain access to the vascular compartment, and binds to IL-1RI receptors on capillaries. This step induces vascular cell adhesion molecule 1 (VCAM1) which promote more monocyte migration and hence increased production of IL-1β. Anti-IL-1β = Canakinumab. Adapted from reference [7].

More than any other cytokine family, the IL-1 family of ligands and receptors – particularly IL-1β isoform – is primarily associated with acute and chronic inflammation^4^. The NOD-like receptor protein 3 (NLRP3) inflammasome activates IL-1β, a process promoted by tissue hypoxia, cholesterol crystals, tissue hypoxia, and arterial flow patterns that are known to promote focal development of atherosclerosis within arteries^5,6^ (Figure 2). IL-1β plays multiple roles in the development and progression of atherothrombotic plaques, including the induction of pro-coagulant activity, the promotion of monocyte and leukocyte adhesion to vascular endothelial cells, and the growth of vascular smooth-muscle cell (Figure 2)^4^.

**Figure 3.**
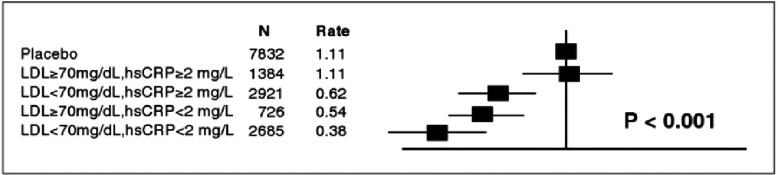
Hazard ratios for incident CV events in the JUPITER trial according to achieved concentrations of LDL-C and Hs-CRP after initiation of rosuvastatin therapy.

Several biomarkers of inflammation – such as high sensitivity CRP (hs-CRP) IL-6, and fibrinogen – have been consistently associated with increased risk of cardiovascular (CV) events, independent of the cholesterol level^8–10^. In a large meta-analysis, the magnitude of CV risk associated with a one standard deviation increase in hs-CRP is at least as large as that associated with a one standard deviation increase in either total cholesterol or blood pressure^11^. Moreover, therapeutic reduction of hs-CRP concentrations in the JUPITER (Justification for the Use of Statins in Prevention: an Intervention Trial Evaluating Rosuvastatin) study was predictive of CV event rates irrespective of the lipid endpoint used^12,13^. Participants who achieved both LDL-C < 1.8 mmol/L and hs-CRP < 2 mg/L in this study had a 65% reduction in CV events (adjusted hazard ratio (HR) 0.35, 95% confidence interval (CI) = 0.23–0.54, *p* < 0.001), compared to a 33% reduction in those who achieved one or neither target (HR 0.67, 95% CI = 0.52-0.87, *p* < 0.001) (Figure 3)^13^. Moreover, a 79% reduction in CV events (HR 0.21, 95% CI 0.09-0.52) has been detected in participants who achieved LDL-C < 1.8 mmol/L and hs-CRP < 1 mg/L^13^.

Whether inhibition of inflammation will reduce CV events is currently a major and unresolved issue. Currently available CV drugs with ant-inflammatory effects, such as aspirin and statins, predominantly exert therapeutic benefits by means of mechanisms other than inflammation. No evidence has shown that reducing vascular inflammation in the absence of concomitant lowering of lipoproteins levels reduces the rates of CV events, thus the inflammatory hypothesis of atherothrombosis has remained unproved.

**Figure 4.**
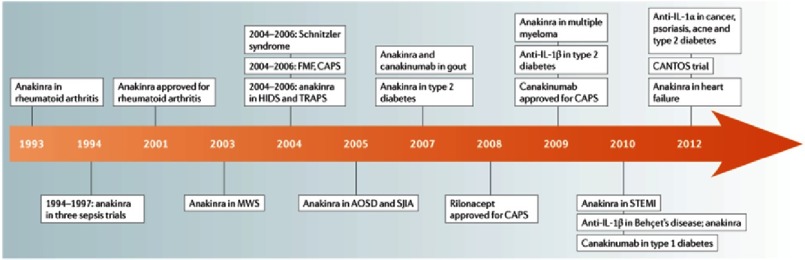
IL-1-blocking agents in various disease states. Adapted from reference [7].

IL-1β has emerged as a therapeutic target for an expanding number of systemic and local auto-inflammatory diseases (Figure 4)^7^. Canakinumab, a fully human monoclonal antibody that neutralizes IL-1β has been approved for clinical use in rheumatologic disorders^14,15^. In addition, plasma levels of IL-6 and Hs-CRP has been significantly reduced with no significant effects on LDL-C and HDL-C levels in patients with diabetes ad high CV risk who were treated with canakinumab^16^. Data from the recently published CANTOS (Canakinumab Anti-inflammatory Thrombosis Outcome Study) provides insights into the potential use of canakinumab to reduce CV risk in patients with atherosclerotic coronary artery disease.

**Figure 5.**
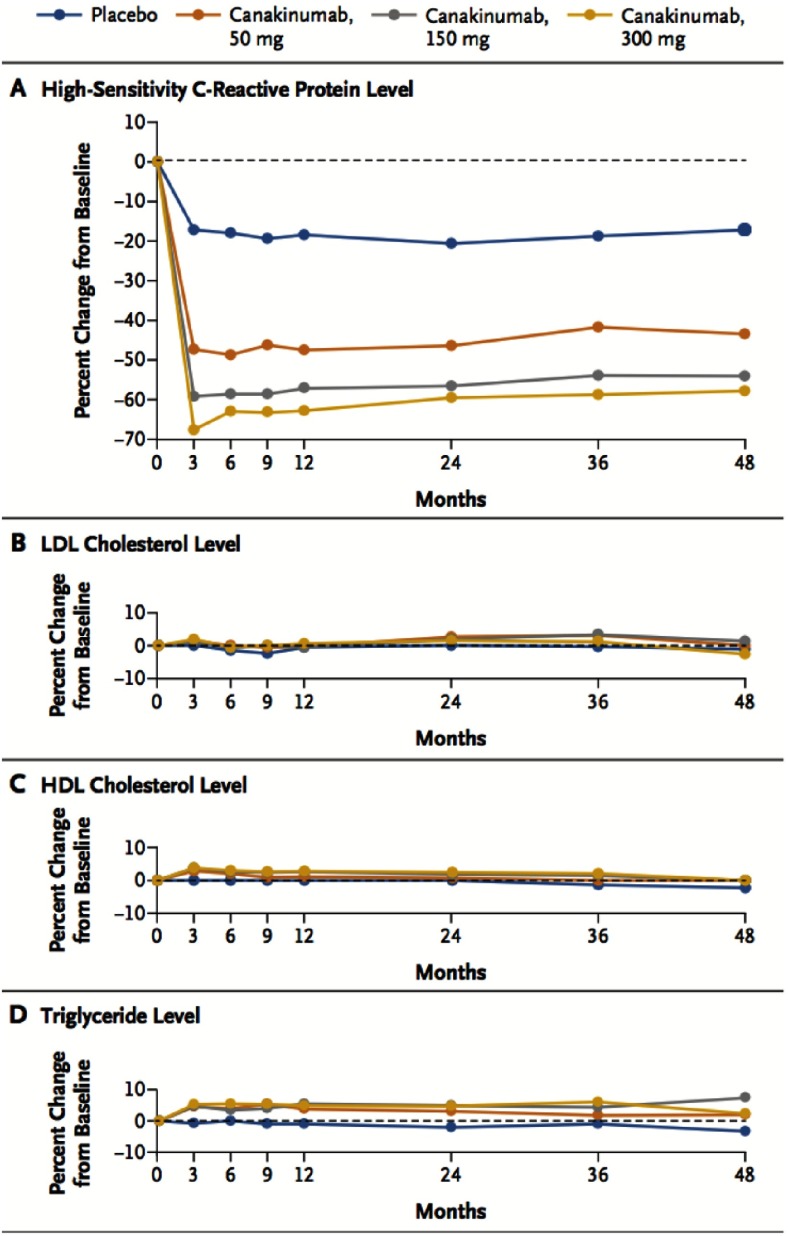
Effects of canakinumab, as compared with placebo, on plasma levels of hs-CRP, LDL-C, HDL-C, and triglycerides.

**Figure 6.**
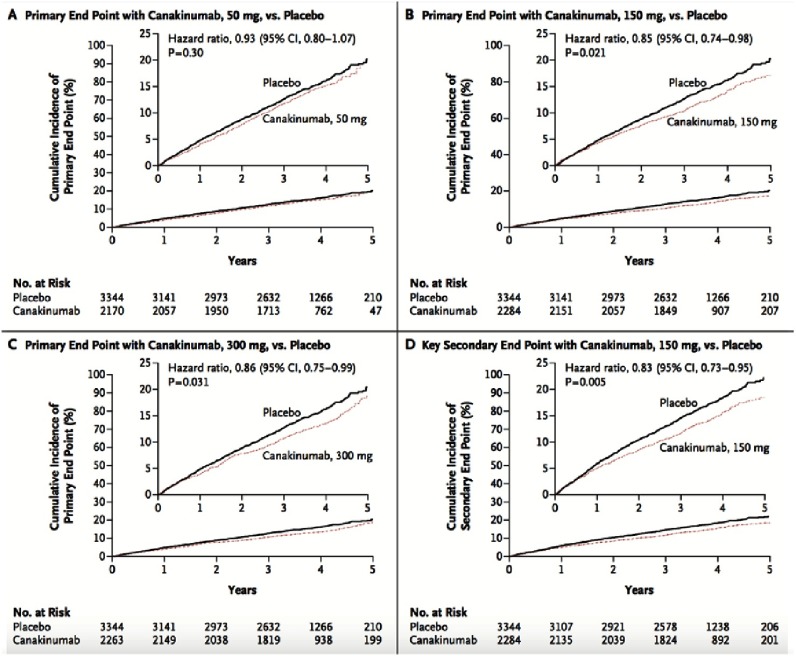
Cumulative incidence of the primary and key secondary end points in the CANTOS study.

## CANTOS study

The CANTOS study was a randomized, double-blind, prospective, controlled clinical trial presented at the European Society of Cardiology (ESC) Congress in Barcelona and published simultaneously in the *New England Journal of Medicine* ^17,18^. The study was designed to assess the efficacy and safety of three doses of canakinumab (50 mg, 150 mg, and 300 mg) administered subcutaneously every 3 months compared to placebo.

A total of 10,061 patients (mean age = 61 years) with previous myocardial infarction (MI) (at least 30 days after MI) and a hs-CRP level ≥ 2 mg/L despite optimal guideline-directed medical treatment. Most participants had undergone previous revascularization procedures (66.7% had undergone percutaneous coronary intervention, and 14% had coronary artery bypass grafting surgery. The trial excluded from enrollment patients with a history of chronic or recurrent infection, previous cancer other than basal-cell skin carcinoma, a suspected or known immunocompromised state, a history or high risk of tuberculosis or disease related to the human immunodeficiency virus, or ongoing use of other systemic anti-inflammatory treatments.

The primary efficacy end point was non-fatal MI, non-fatal stroke, or CV death. The secondary efficacy end points included, (1) the components of the primary end point as well as hospitalization for unstable angina that led to urgent revascularization, (2) the incidence of new-onset type 2 diabetes among patients with prediabetes at randomization (data was not available yet), (3) death from any cause (4) the composite of non-fatal MI, any non-fatal stroke, or death from any cause. The components of these end points were adjudicated by an end-point adjudication committee, whose members were unaware of the trial-group assignments.

At 48 months, canakinumab groups had achieved greater reduction from baseline in the hs-CRP level than in the placebo group. No significant reduction in lipid levels had been detected in the canakinumab groups compared to placebo (Figure 5). At a median follow-up of 3.7 years, the incidence rate for the primary end point was significantly lower in the canakinumab 150 mg group (HR was 0.85, 95% CI, 0.74 to 0.98; *P* = 0.021) and the 300 mg group (0.86, 95% CI = 0.75 to 0.99; *P* = 0.031) compared to placebo. No significant effect was observed with regard to the primary end point in the 50-mg group (Figure 6).

The 150-mg dose (but not other doses), met the prespecified multiplicity-adjusted threshold for statistical significance for the primary end point and the secondary end point. Neutropenia, and deaths due to infection, was more common in the pooled canakinumab groups than placebo group. Thrombocytopenia was also more common in the canakinumab groups than placebo group, but no significant difference in the incidence of hemorrhage was observed. There was no significant difference in all-cause mortality between canakinumab groups and placebo group (*P* = 0.31).

## Discussion

Atherosclerosis is no longer considered solely a disorder of subintimal deposition of modified LDL particles in the arterial wall. Rather, it is a chronic inflammatory disorder. In the past decades, the concept of the involvement of inflammation in atherosclerosis has spurred the discovery and adoption of inflammatory biomarkers for CV risk prediction, and also the development of anti-inflammatory therapies that can modulate the inflammatory cascade and modify the inflammasome in order to reduce future CV events and residual risk in high risk patients.

Although hypothesis generating, existing analyses of inflammatory biomarkers within statin trials can not definitively evaluate the inflammatory hypothesis of atherosclerosis because of the predominant lipid lowering effect of statins. Similarly, the observation that aspirin reduces CV risk with a greater magnitude when CRP levels are high does not definitively address this issue^19^. CANTOS is the first study to directly test the inflammatory hypothesis of atherosclerosis. In this study, the anti-inflammatory drug (canakinumab) in 150-mg and 300 mg doses, four times a year, achieved a 40% relative risk reduction (RRR) in plasma CRP and IL-6 levels, in addition to a 15% RRR in the incidence of MI, stroke, or CV death. No change in plasma LDL-C, HDL-C, or triglycerides levels have been demonstrated. Interestingly, the canakinumab group had a 30% RRR in the endpoint of any need for bypass surgery or revascularization at any point in the trial, which may be attributed to slowing the progression of atherosclerosis. On the other hand, there was significantly higher rate of fatal infection and sepsis in patients received canakinumab compared to placebo ( *p* = 0.02).

Despite optimal medical therapy in the placebo group, which included statin therapy in more than 90% of patients that achieved a baseline LDL-C level around 80 mg/dl, the rate of CV events at 5 years is relatively high and reaches up to 25%, which raise the importance of developing new classes of drugs that can decrease future CV events in those patients. It is worth noting that the patients included in this study could be also candidates for the recently approved pro-protein convertase subtilisin Kexin (PCSK9) inhibitors, however the high baseline plasma CRP level in those patients (median CRP 4.1 mg/L) may predict a more beneficial effect from an anti-inflammatory drug rather than a lipid lowering agent.

Cancer mortality was also significantly lower among patients assigned to receive canakinumab than among those in the placebo group, a finding that is consistent with experimental data relating IL-1 to the progression and invasiveness of certain tumors, particularly lung cancer.

Despite the encouraging results of this trial, two important points needs to be raised. Firstly, canakinumab had a significant, but modest, effect regarding reduction of non-fatal CV events, however it has no effect on CV or all-cause mortality. Secondly, it was associated with increased mortality due to infection and sepsis. Thus, the net benefit needs to be properly addressed in the future studies that evaluate the potential benefit of the anti-inflammatory therapies, that either inhibit NLRP3 function, or downstream ILs signaling, taking in consideration that the current cost – $200,000 per year – may be prohibitive for its general use in such common disease^20^.

## What have we learned?

The development of new biologic compounds, like IL-1β and PCSK9 inhibitors, helps to modify the disease and improve outcome in high risk patients. The magnitude of effect on CV events with canakinumab (given every 3 months) was similar to that associated with the already FDA approved PCSK9 inhibitors (given every 2 to 4 weeks) in absence of any significant effect on lipoproteins levels^21^. The CANTOS study is an extremely exciting proof of concept clinical trial that opens up a new way of thinking about halting the progression of atherosclerosis and reducing residual CV risk. However, considerably more research is needed for such anti-inflammatory therapies in order to change clinical practice and to reach the clinic. The results of the ongoing Cardiovascular Inflammation Reduction Trial (CIRT) – examining the efficacy and safety of low-dose methotrexate in patients with a previous MI who have diabetes or the metabolic syndrome - are eagerly awaited.^22^
